# Closed Formulas for Some New Degree Based Topological Descriptors Using M-polynomial and Boron Triangular Nanotube

**DOI:** 10.3389/fchem.2020.613873

**Published:** 2021-02-03

**Authors:** Dong Yun Shin, Sabir Hussain, Farkhanda Afzal, Choonkil Park, Deeba Afzal, Mohammad R. Farahani

**Affiliations:** ^1^Department of Mathematics, University of Seoul, Seoul, South Korea; ^2^Department of Mathematics and Statistics, The University of Lahore, Lahore, Pakistan; ^3^Department of Humanities and Basic Sciences, Military College of Signals, National University of Science and Technology, Islamabad, Pakistan; ^4^Research Institute for Natural Sciences, Hanyang University, Seoul, South Korea; ^5^Department of Mathematics, Iran University of Science and Technology, Tehran, Iran

**Keywords:** graph, chemical graph theory, M-polynomial, boron triangular nanotube, topological indices

## Abstract

In this article, we provide new formulas to compute the reduced reciprocal randi*ć* index, Arithmetic geometric_1_ index, *SK* index, *SK*_1_ index, *SK*_2_ index, edge version of the first zagreb index, sum connectivity index, general sum connectivity index, and the forgotten index using the M-polynomial and finding these topological indices for a boron triangular nanotube. We also elaborate the results with graphical representations.

## 1. Introduction

A chemical molecular structure is composed of atoms that join together with chemical bonds. This molecular structure is responsible for the chemical, physical, and biological properties of the chemical compound. A chemical graph theory is an important field of science, in which we study the formation and behavior of a chemical structure with the help of graph theory tools.

In graph theory a set of points is referred as graph *G*. These points are known as vertices. An edge is a line joining the two vertices. The number of edges that coincide at a vertex is considered to be the degree of the vertex and is represented as *d*_*j*_ and the degree of the edge is defined as *d*_*jk*_ = *d*_*j*_ + *d*_*k*_ − 2. *E*(*G*) represents the set of edges and *V*(*G*) shows the set of vertices. In the chemical graph, an atom of the molecule is represented with the vertex of the graph, and the bond is considered as an edge.

There are many uses of chemical graph theory in different subjects, such as quantum chemistry, computer sciences, biology, stereochemistry, and engineering, which is explained by Gutman and Trinajstić (1972), Balaban (1985), Trinajstiéc (1992), Shirinivas et al. (2010), and Vergniory et al. (2017). With the help of chemical graph theory techniques, we convert a chemical molecule into a real number, which is referred to as a topological index and the molecular structure is examined through the topological indices. In this study, we try to develop mathematical methods for the calculation of topological indices. With the help of topological indices, Hosamani et al. (2017) studied the different physical properties like the molar volume, boiling points, and molar refraction, of the molecular structure. Rouvray (1986) and Ramakrishnan et al. (2013) describe the biological behavior, such as nutritive, stimulation of cell growth, toxicity, and pH regulation, of the chemical species, which are also characterized through topological indices.

These topological indices are widely used in chemical graph theory to explain the different properties of the chemical structure. The carbon-hydrogen bond is not considered during the computation of topological indices because this bond does not have a serious effect on the properties of the chemical compound. During the QSAR and QSPR analysis, topological indices are widely used.

Gutman et al. (2014) formulated a reduced reciprocal randić index defined as $RRR=\underset{jk\in E\left(G\right)}{\sum }\sqrt{\left(d_{j}-1\right)\left(d_{k}-1\right)}$. Shigehalli and Kanabur (2015) presented the arithmetic geometric_1_ index defined as $AG_{1}=\underset{jk\in E\left(G\right)}{\sum }\frac{d_{j}+d_{k}}{2\sqrt{d_{j}\cdot d_{k}}}$. Shigehalli and Kanabur (2016) also introduced the new indices defined as *SK* index = $\underset{jk\in E\left(G\right)}{\sum }\frac{d_{j}+d_{k}}{2}$, *SK*_1_ index = $\underset{jk\in E\left(G\right)}{\sum }\frac{d_{j}\cdot d_{k}}{2}$, and *SK*_2_ index = $\underset{jk\in E\left(G\right)}{\sum }(\frac{d_{j}+d_{k}}{2}{)}^{2}$. Miličević et al. (2004) presented the first Zagreb index in term of edge degree defined as $EM_{1}=\underset{jk\in E\left(G\right)}{\sum }{\left(d_{jk}\right)}^{2}$. Du et al. (2011) formulated the general sum-connectivity index defined as $SCI=\underset{jk\in E\left(G\right)}{\sum }\frac{1}{\sqrt{d_{j}+d_{k}}}$ and $SCI_{\lambda }=\underset{jk\in E\left(G\right)}{\sum }(d_{j}+d_{k}{)}^{\lambda }$. Gutman and Furtula (2015) presented the forgotten index represented with *F* and defined as $F=\underset{jk\in E\left(G\right)}{\sum }(d_{j}^{2}+d_{k}^{2})$.

## 2. Boron Triangular Nanotube

The analysis of a chemical molecular structure smaller than 100 nm is known as nanotechnology. Nanomaterials have many applications in different fields of nanoscience. The boron triangular nanotube *BTnt_lq_* is a well-known structure in nanomaterials with a wide range of applications in medicine, electronics, and computers as discussed by Menuel (2010), Bezugly et al. (2011), and Liu et al. (2018). With the help of this, experts are expected to revolutionize the world. The formation of the boron triangular nanotube is formed by a 2-D boron triangular nanosheet consisting of *l* rows and *q* columns. The first and the last column of the 2-D boron triangular nanosheet are connected to form a boron triangular nanotube.

**Figure 1:**
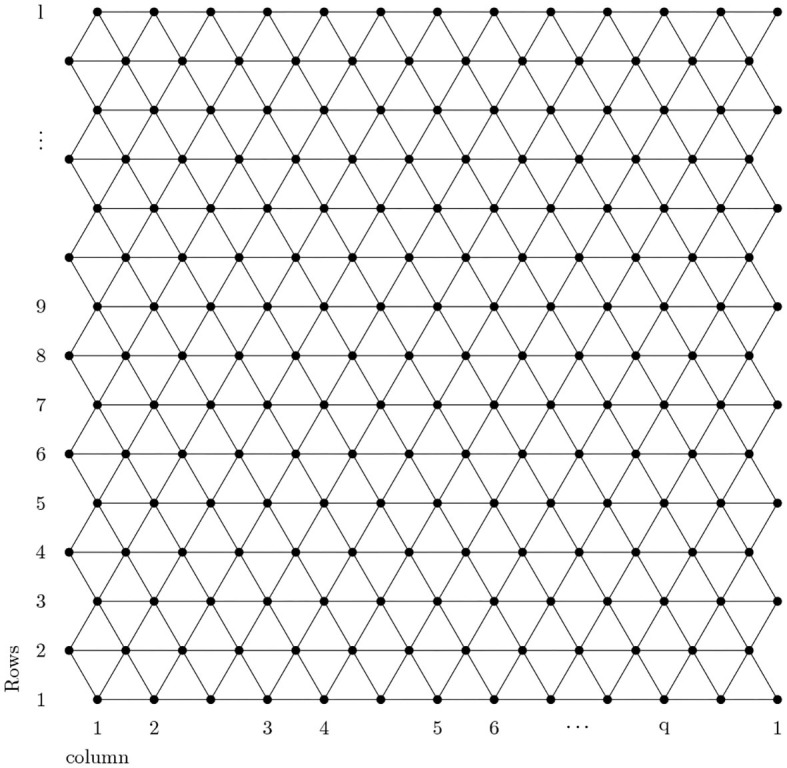
Boron triangular nanosheet.

**Figure 2:**
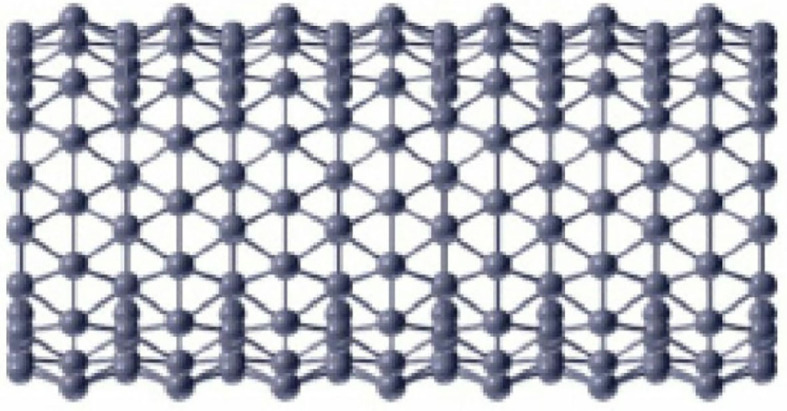
Boron triangular nanotube (*BTnt_lq_*).

A detailed analysis of Figures 1, 2 shows that there are only two types of vertex present in *BTnt_lq_*. One which has a degree of four and the other has a degree of six. Now count the edges with smaller dimension and then by generalization and we obtain Table 1.

**Table 1:** Edge partition of boron triangular nanotube (*BTnt_lq_*).

**(*d*_*u*_, *d*_υ_)**	**Number of edges**
(4,4)	3*q*
(4,6)	6*q*
(6,6)	$\frac{q}{2}\left(9l-24\right)$

## 3. M-Polynomial

An algebraic polynomial has the ability to elaborate the chemical molecular structure. M-polynomial is such a polynomial that represents the graph. Deutsch and Klawzar (2015) formulated the M-polynomial and defined as $M\left(G,x,y\right)=\underset{\delta \leq u\leq \upsilon \leq \Delta }{\sum }m_{uv}\left(G\right)x^{u}y^{\upsilon }$. Where δ represents the minimum degree of the vertex belonging to the vertex set *V*(*G*), Δ represents the maximum degree of the vertex belonging to the vertex set *V*(*G*) and *m*_υ*v*_(*G*) is the total number of edges *jk* ∈ *E*(*G*) such that {*d*_*j*_, *d*_*k*_} = {*u*, υ}.

Deutsch and Klawzar (2015) introduced M-polynomial and in the same article nine topological indices closed formulas are given via m-polynomial. Some indices are calculated by Munir et al. (2016). In the past, Kang et al. (2018), Afzal et al. (2020a,b), Cancan et al. (2020a,b), and Khalaf et al. (2020) used these formulas to compute topological indices via M-polynomial and a lot of work has been done in this area. One more set of nine topological indices has been computed by Afzal et al. (2020c). The F-index is also computed by Mondal et al. (2020). In this article, we formulated a new set of nine topological indices shown in Table 2. These formulas are used to compute the topological indices via M-polynomial for the chemical structure. This formulation is a useful achievement in the field the topological indices and opens a new research field. We apply the newly formulated indices on *BTnt_lq_*.

**Table 2:** Topological indices derive from *M*(*BTnt*_*lq*_; *x, y*).

**Topological index**		**Derivation from *M(*BTnt*_*lq*_; *x, y*) = *g*(*x, y*)***
*RRR*[*BTnt*_*lq*_]	=	$D_{x}^{\frac{1}{2}}D_{y}^{\frac{1}{2}}Q_{y\left(-1\right)}Q_{x\left(-1\right)}{\left[g\left(x,y\right)\right]}_{x=y=1}$
*AG*_1_[*BTnt*_*lq*_]	=	$\frac{1}{2}D_{x}JS_{x}^{\frac{1}{2}}S_{y}^{\frac{1}{2}}{\left[g\left(x,y\right)\right]}_{x=1}$
*SK*[*BTnt*_*lq*_]	=	$\frac{1}{2}\left(D_{x}+D_{y}\right){\left[g\left(x,y\right)\right]}_{x=y=1}$
*SK*_1_[*BTnt*_*lq*_]	=	$\frac{1}{2}D_{x}D_{y}{\left[g\left(x,y\right)\right]}_{x=y=1}$
*Sk*_2_[*BTnt*_*lq*_]	=	$\frac{1}{4}D_{x}^{2}J{\left[g\left(x,y\right)\right]}_{x=y=1}$
*EM*_1_[*BTnt*_*lq*_]	=	$D_{x}^{2}Q_{x\left(-2\right)}J{\left[g\left(x,y\right)\right]}_{x=1}$
*SCI*[*BTnt*_*lq*_]	=	$S_{x}^{\frac{1}{2}}J{\left[g\left(x,y\right)\right]}_{x=1}$
*SCI*_λ_[*BTnt*_*lq*_]	=	$D_{x}^{\lambda }J{\left[g\left(x,y\right)\right]}_{x=1}$
*F*[*BTnt*_*lq*_]	=	$\left(D_{x}^{2}+D_{y}^{2}\right){\left[g\left(x,y\right)\right]}_{x=1}$

Where the operator used are defined as

$D_{x}g\left(x,y\right)=x\frac{\partial \left(g\left(x,y\right)\right)}{\partial x},$ $D_{y}g\left(x,y\right)=y\frac{\partial \left(g\left(x,y\right)\right)}{\partial y},$ *Jg*(*x, y*) = *g*(*x, x*), $Q_{x\left(\alpha \right)}g\left(x,y\right)=x^{\alpha }g\left(x,y\right),$

$D_{x}^{\frac{1}{2}}g\left(x,y\right)=\sqrt{x\frac{\partial \left(g\left(x,y\right)\right)}{\partial x}}\cdot \sqrt{g\left(x,y\right)},$      $D_{y}^{\frac{1}{2}}g\left(x,y\right)=\sqrt{y\frac{\partial \left(g\left(x,y\right)\right)}{\partial y}}\cdot \sqrt{g\left(x,y\right)},$

$S_{x}^{\frac{1}{2}}g\left(x,y\right)=\sqrt{\overset{x}{\underset{0}{\int }}\frac{g\left(t,y\right)}{t}dt}\cdot \sqrt{g\left(x,y\right)},$     $S_{y}^{\frac{1}{2}}g\left(x,y\right)=\sqrt{\overset{y}{\underset{0}{\int }}\frac{g\left(x,t\right)}{t}dt}\cdot \sqrt{g\left(x,y\right)}$.

## 4. M-Polynomial of Boron Triangular Nanotube

**Theorem 4.1**. *Let BTnt*_*lq*_ *be a Boron triangular nanotube where *lq* is the dimension of the *BTnt*_*lq*_ then M-polynomial of *BTnt*_*lq*_ is*

$$M\left[BTnt_{lq};x,y\right]=3qx^{4}y^{4}+6qx^{4}y^{6}+\frac{q}{2}\left(9l-24\right)x^{6}y^{6}.$$

*Proof*. Let *BTnt*_*lq*_ be a boron triangular nanotube then by using Figures 1, 2 and Table 1 the edge partition of boron triangular nanotube is consisting of three type of sets. The first edge partition represented with *E*_1_ contains 3*q* edges, in which *d*_*j*_ and *d*_*k*_ have the same value equal to 4. The second edge partition referred to as *E*_2_ consists of 6*q* edges *jk*, in which the value of *d*_*j*_ is 4 and value of *d*_*k*_ is 6. The third edge partition named as *E*_3_ contains $\frac{q}{2}\left(9l-24\right)$ edges *jk*, in which *d*_*j*_ = *d*_*k*_ = 6. Now, by using the definition of M-polynomial, we have

$$\begin{array}{l} M\left(BTnt_{lq};x,y\right)=\underset{\delta \leq u\leq \upsilon \leq \Delta }{\sum }m_{u\upsilon }\left(B_{\alpha }NT_{mn}\right)x^{u}y^{\upsilon }\\ \;=\underset{4\leq u\leq v\leq 6}{\sum }m_{uv}\left(BTnt_{lq}\right)x^{u}y^{v},\\ M\left(BTnt_{lq};x,y\right)=\underset{4\leq 4}{\sum }m_{44}\left(BTnt_{lq}\right)x^{4}y^{4}+\underset{4\leq 6}{\sum }m_{46}\left(BTnt_{lq}\right)x^{4}y^{6}\\ \;+\underset{6\leq 6}{\sum }m_{66}\left(BTnt_{lq}\right)x^{6}y^{6},\\ M\left(BTnt_{lq};x,y\right)=|E_{4,4}|x^{4}y^{4}+|E_{4,6}|x^{4}y^{6}+|E_{6,6}|x^{6}y^{6},\\ M\left[BTnt_{lq};x,y\right]=3qx^{4}y^{4}+6qx^{4}y^{6}+\frac{q}{2}\left(9l-24\right)x^{6}y^{6}. \end{array}$$

**Figure 3:**
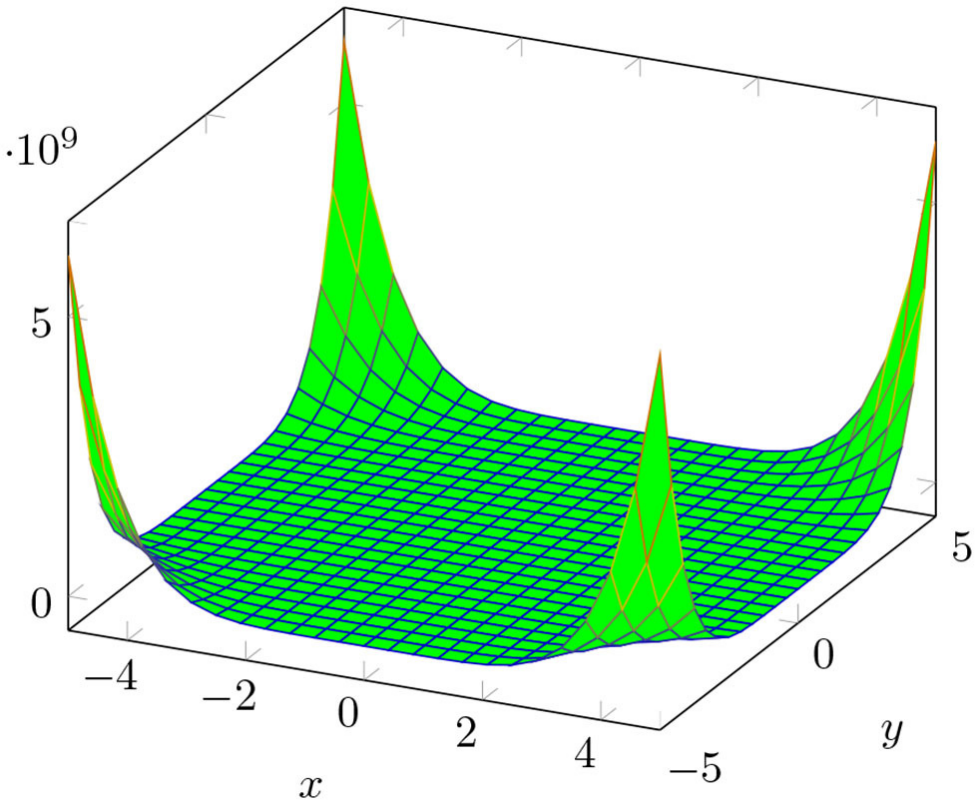
3D plots of M-polynomial of boron triangular nanotube *BTnt_lq_* for *l* = *q* = 4.

The plot of *M*[*BTnt*_*lq*_; *x, y*] is shown in Figure 3.

## 5. Topological Indices of Boron Triangular Nanotube

**Theorem 5.1**. *Let *BTnt*_*lq*_ be a boron triangular nanotube and*

$$M\left[BTnt_{lq};x,y\right]\;=\;3qx^{4}y^{4}+6qx^{4}y^{6}+\frac{q}{2}\left(9l-24\right)x^{6}y^{6},$$

then

1. $RRR\left[{BTnt}_{lq}\right]\;=\;\frac{45}{2}ql+\left(6\sqrt{15}-51\right)q,$
2. $AG_{1}\left[{BTnt}_{lq}\right]\;=\;\frac{9}{2}ql+\left(\frac{5\sqrt{6}}{2}-9\right)q,$
3. *SK*[*BTnt_lq_*] = 27*ql* − 30*q*,
4. *SK*_1_[*BTnt_lq_*] = 81*ql* − 120*q*,
5. *SK*_2_[*BTnt_lq_*] = 162*ql* − 234*q*,
6. *EM*_1_[*BTnt_lq_*] = 450*ql* − 708*q*,
7. $SCI\left[{BTnt}_{lq}\right]\;=\;\frac{3\sqrt{3}}{4}ql+\left(\frac{3\sqrt{2}}{4}+\frac{3\sqrt{10}}{5}-2\sqrt{3}\right)q,$
8. $SCI_{\lambda }\left[{BTnt}_{lq}\right]\;=\;\frac{9}{2}{\left(12\right)}^{\lambda }ql+\left\{3{\left(8\right)}^{\lambda }+6{\left(10\right)}^{\lambda }-24{\left(12\right)}^{\lambda }\right\}q,$
9. *F*[*BTnt_lq_*] = 324*ql* − 456*q*.

*Proof*. Let M[*BTnt_lq_*; x,y] = g(x,y)

$$\begin{array}{l} \;Q_{x\left(-1\right)}g\left(x,y\right)=3qx^{3}y^{4}+6qx^{3}y^{6}+\frac{q}{2}\left(9l-24\right)x^{5}y^{6},\\ \;Q_{y\left(-1\right)}Q_{x\left(-1\right)}g\left(x,y\right)=3qx^{3}y^{3}+6qx^{3}y^{5}+\frac{q}{2}\left(9l-24\right)x^{5}y^{5},\\ \;D_{y}^{\frac{1}{2}}Q_{y\left(-1\right)}Q_{x\left(-1\right)}g\left(x,y\right)=3\sqrt{3}qx^{3}y^{3}+6\sqrt{5}qx^{3}y^{5}\\ \;+\frac{\sqrt{5}}{2}q\left(9l-24\right)x^{5}y^{5},\\ D_{x}^{\frac{1}{2}}D_{y}^{\frac{1}{2}}Q_{y\left(-1\right)}Q_{x\left(-1\right)}g\left(x,y\right)=9qx^{3}y^{3}+6\sqrt{15}qx^{3}y^{5}\\ \;+\frac{5}{2}q\left(9l-24\right)x^{5}y^{5},\\ \;S_{y}^{\frac{1}{2}}g\left(x,y\right)\;=\;\frac{3}{2}qx^{4}y^{4}+\sqrt{6}qx^{4}y^{6}+\frac{q}{2\sqrt{6}}\left(9l-24\right)x^{6}y^{6},\\ \;S_{x}^{\frac{1}{2}}S_{y}^{\frac{1}{2}}g\left(x,y\right)\;=\;\frac{3}{4}qx^{4}y^{4}+\frac{\sqrt{6}}{2}qx^{4}y^{6}+\frac{q}{12}\left(9l-24\right)x^{6}y^{6},\\ \;JS_{x}^{\frac{1}{2}}S_{y}^{\frac{1}{2}}g\left(x,y\right)\;=\;\frac{3}{4}qx^{8}+\frac{\sqrt{6}}{2}qx^{10}+\frac{q}{12}\left(9l-24\right)x^{12},\\ \;D_{x}JS_{x}^{\frac{1}{2}}S_{y}^{\frac{1}{2}}g\left(x,y\right)=\;6qx^{8}+5\sqrt{6}qx^{10}+q\left(9l-24\right)x^{12},\\ \;\frac{1}{2}D_{x}JS_{x}^{\frac{1}{2}}S_{y}^{\frac{1}{2}}g\left(x,y\right)\;=\;3qx^{8}+\frac{5\sqrt{6}}{2}qx^{10}+\frac{q}{2}\left(9l-24\right)x^{12},\\ \;D_{x}g\left(x,y\right)\;=\;12qx^{4}y^{4}+24qx^{4}y^{6}+3q\left(9l-24\right)x^{6}y^{6},\\ \;D_{y}g\left(x,y\right)\;=\;12qx^{4}y^{4}+36qx^{4}y^{6}+3q\left(9l-24\right)x^{6}y^{6},\\ \;\left(D_{x}+D_{y}\right)g\left(x,y\right)\;=\;24qx^{4}y^{4}+60qx^{4}y^{6}+6q\left(9l-24\right)x^{6}y^{6},\\ \;\frac{1}{2}\left(D_{x}+D_{y}\right)g\left(x,y\right)\;=\;12qx^{4}y^{4}+30qx^{4}y^{6}+3q\left(9l-24\right)x^{6}y^{6},\\ \;D_{x}D_{y}g\left(x,y\right)\;=\;48qx^{4}y^{4}+144qx^{4}y^{6}+18q\left(9l-24\right)x^{6}y^{6},\\ \;\frac{1}{2}\left(D_{x}D_{y}\right)g\left(x,y\right)\;=\;24qx^{4}y^{4}+72qx^{4}y^{6}+9q\left(9l-24\right)x^{6}y^{6},\\ \;Jg\left(x,y\right)\;=\;3qx^{8}+6qx^{10}+\frac{q}{2}\left(9l-24\right)x^{12},\\ \;D_{x}^{2}Jg\left(x,y\right)\;=\;192qx^{8}+600qx^{10}+72\left(9l-24\right)x^{12},\\ \;\frac{1}{4}D_{x}^{2}Jg\left(x,y\right)\;=\;48qx^{8}+150qx^{10}+18\left(9l-24\right)x^{12},\\ \;Q_{x\left(-2\right)}Jg\left(x,y\right)\;=\;3qx^{6}+6qx^{8}+\frac{q}{2}\left(9l-24\right)x^{10},\\ \;D_{x}^{2}Q_{x\left(-2\right)}Jg\left(x,y\right)\;=\;108qx^{6}+384qx^{8}+50q\left(9l-24\right)x^{10},\\ \;S_{x}^{\frac{1}{2}}Jg\left(x,y\right)\;=\;\frac{3}{2\sqrt{2}}qx^{8}+\frac{6}{\sqrt{10}}qx^{10}+\frac{q}{4\sqrt{3}}\left(9l-24\right)x^{12},\\ \;D_{x}^{\lambda }Jg\left(x,y\right)\;=\;3{\left(8\right)}^{\lambda }qx^{8}+6{\left(10\right)}^{\lambda }qx^{10}\\ \;+\frac{q}{2}{\left(12\right)}^{\lambda }\left(9l-24\right)x^{12},\\ \;D_{x}^{2}g\left(x,y\right)\;=\;48qx^{4}y^{4}+96qx^{4}y^{6}+18q\left(9l-24\right)x^{6}y^{6},\\ \;D_{y}^{2}g\left(x,y\right)\;=\;48qx^{4}y^{4}+216qx^{4}y^{6}+18q\left(9l-24\right)x^{6}y^{6},\\ \;\left(D_{x}^{2}+D_{y}^{2}\right)g\left(x,y\right)\;=\;96qx^{4}y^{4}+312qx^{4}y^{6}+36q\left(9l-24\right)x^{6}y^{6}. \end{array}$$

1. $RRR\left[BTnt_{lq}\right]\;=\;D_{x}^{\frac{1}{2}}D_{y}^{\frac{1}{2}}Q_{y\left(-1\right)}Q_{x\left(-1\right)}{\left[g\left(x,y\right)\right]}_{x=y=1},$
  $RRR\left[{BTnt}_{lq}\right]\;=\;\frac{45}{2}ql+\left(6\sqrt{15}-51\right)q.$
2. $AG_{1}\left[BTnt_{lq}\right]\;=\;\frac{1}{2}D_{x}JS_{x}^{\frac{1}{2}}S_{y}^{\frac{1}{2}}{\left[g\left(x,y\right)\right]}_{x=1},$
  $AG_{1}\left[{BTnt}_{lq}\right]\;=\;\frac{9}{2}ql+\left(\frac{5\sqrt{6}}{2}-9\right)q.$
3. $SK\left[BTnt_{lq}\right]\;=\;\frac{1}{2}\left(D_{x}+D_{y}\right){\left[g\left(x,y\right)\right]}_{x=y=1},$
  *SK*[*BTnt_lq_*] = 27*ql* − 30*q*.
4. $SK_{1}\left[BTnt_{lq}\right]\;=\;\frac{1}{2}D_{x}D_{y}{\left[g\left(x,y\right)\right]}_{x=y=1},$
  *SK*_1_[*BTnt_lq_*] = 81*ql* − 120*q*.
5. $Sk_{2}\left[BTnt_{lq}\right]\;=\;\frac{1}{4}D_{x}^{2}J{\left[g\left(x,y\right)\right]}_{x=y=1},$
  *SK*_2_[*BTnt_lq_*] = 162*ql* − 234*q*.
6. $EM_{1}\left[BTnt_{lq}\right]\;=\;D_{x}^{2}Q_{x\left(-2\right)}J{\left[g\left(x,y\right)\right]}_{x=1},$
  *EM*_1_[*BTnt_lq_*] = 450*ql* − 708*q*.
7. $SCI\left[BTnt_{lq}\right]\;=\;S_{x}^{\frac{1}{2}}J{\left[g\left(x,y\right)\right]}_{x=1},$
  $SCI\left[{BTnt}_{lq}\right]\;=\;\frac{3\sqrt{3}}{4}ql+\left(\frac{3\sqrt{2}}{4}+\frac{3\sqrt{10}}{5}-2\sqrt{3}\right)q.$
8. $SCI_{\lambda }\left[BTnt_{lq}\right]\;=\;D_{x}^{\lambda }J{\left[g\left(x,y\right)\right]}_{x=1},$
  $SCI_{\lambda }\left[{BTnt}_{lq}\right]\;=\;\frac{9}{2}12^{\lambda }ql+\left\{3{\left(8\right)}^{\lambda }+6{\left(10\right)}^{\lambda }-24{\left(12\right)}^{\lambda }\right\}q.$
9. $F\left[BTnt_{lq}\right]\;=\;\left(D_{x}^{2}+D_{y}^{2}\right){\left[g\left(x,y\right)\right]}_{x=1},$
  *F*[*BTnt_lq_*] = 324*ql* − 456*q*.

**Figure 4:**
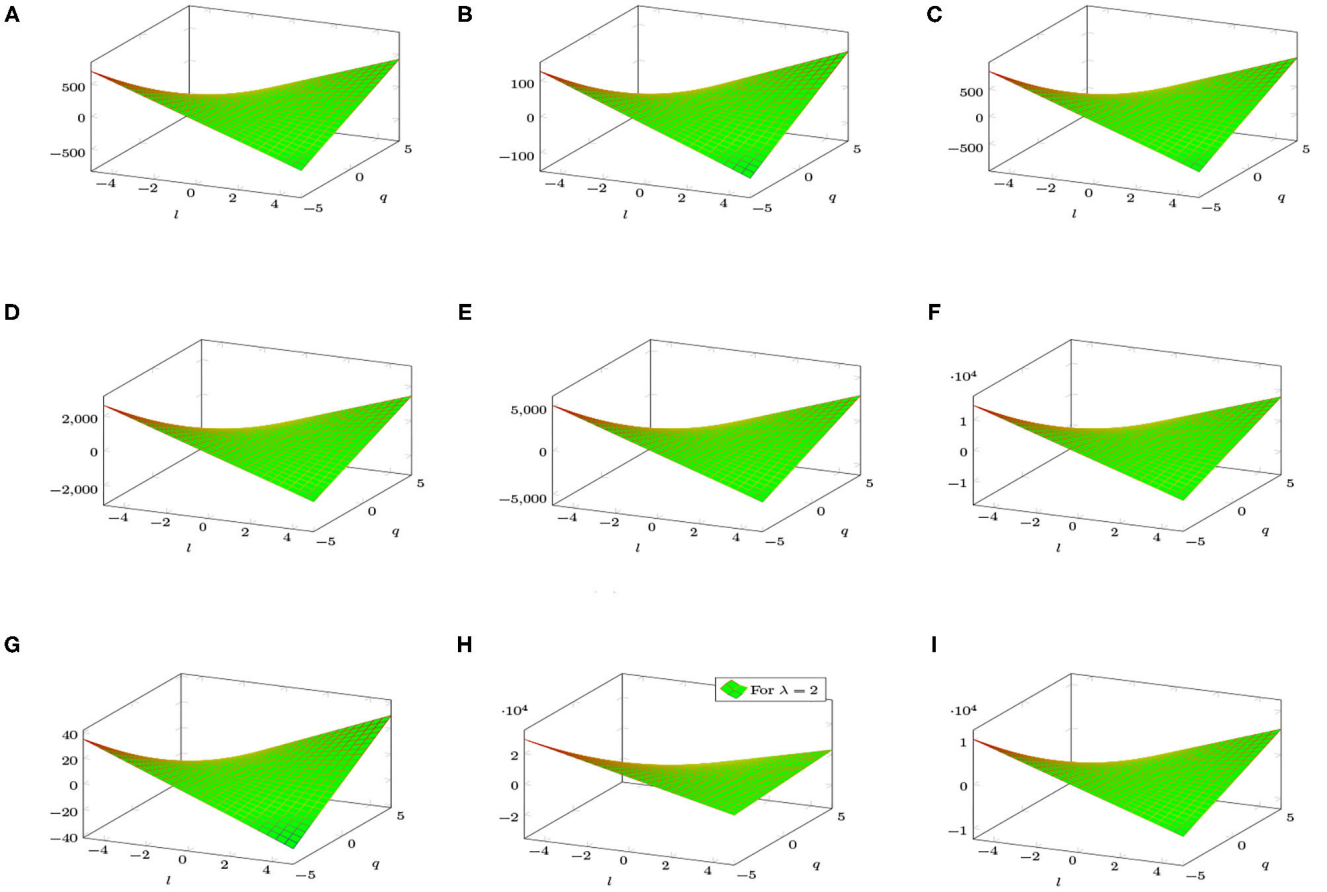
3D plots of topological indices of *BTnt_lq_*. **(A)** Reduced reciprocal randić index. **(B)** Arithmetic geometric_1_ index. **(C)** SK index. **(D)** SK_1_ index. **(E)** SK_2_ index. **(F)** Edge version of first Zagreb index. **(G)** Sum connectivity index. **(H)** General sum connectivity index. **(I)** Forgotten index.

Figure 4 shows a graphical analysis of topological indices of *BTnt_lq_*. With the help of these graphs, we observe the behavior of the topological indices regarding the different parameters involved. These visualizations are shown to be identical but have different gradients.

## 6. Conclusion

The formulation of new formulas, placed in Table 2, to compute the topological indices for the molecular structure via M-polynomial lead to a new era in the computational field. In this research work, we compute *M*(*BTnt_lq_*; *x, y*) and with the help of this polynomial, we find the various topological invariants given in Table 2. We also presented the graphical presentation of M-Polynomial and topological indices. This visualization helps us to understand results against parameters.

## Data Availability Statement

The original contributions presented in the study are included in the article/supplementary material, further inquiries can be directed to the corresponding author/s.

## Author Contributions

DS: supervision, resources, and funding. SH: writing original draft, computations, and graphs. FA: editing, proof reading, and methodology. CP: investigation, validation, and funding. DA: conceptualization, supervision, and programming. MF: resources, proof reading, and validation. All authors contributed to the article and approved the submitted version.

## Conflict of Interest

The authors declare that the research was conducted in the absence of any commercial or financial relationships that could be construed as a potential conflict of interest.
